# Implementing a Longitudinal Adolescent Transition of Care Curriculum: Identifying Comfort and Barriers Among Residents

**DOI:** 10.7759/cureus.29394

**Published:** 2022-09-21

**Authors:** Mihika Sathe, Alissa S Werzen, Natalie Davis, Leah S Millstein

**Affiliations:** 1 Internal Medicine-Pediatrics, University of Maryland Medical Center, Baltimore, USA; 2 Pediatrics, University of Maryland School of Medicine, Baltimore, USA; 3 Internal Medicine-Pediatrics, University of Maryland School of Medicine, Baltimore, USA

**Keywords:** transition to adults, transition of care, resident clinic, peds, med peds, medical education curriculum, medical resident education, adolescent medicine, adolescent and young adults

## Abstract

Amid growing recognition of the importance of transitioning adolescents and young adults (AYA) from pediatric- to adult-oriented health care systems, residency programs are being tasked with educating residents on best transition practices. However, consensus on how to approach training residents in transition of care (TOC) is limited. Our academic residency program therefore created and implemented a TOC of AYA curriculum for pediatric residents in an effort to increase provider knowledge and comfort with this topic. Three classes of post-graduate year one (PGY1) pediatric residents participated in this curriculum from 2017-2019 (n=35) and subsequently completed a problem-based learning (PBL) exercise in a primary care clinic with adolescent patients based on core goals in transitioning AYA. Residents completed pre-PBL and post-PBL surveys quantifying provider comfort in several aspects of the transition process. The majority of residents (94%) identified the PBL exercise as being useful, with no significant difference between classes. Eighty-nine percent (n=31) identified 1) earlier introduction of TOC and/or 2) incorporation of TOC discussions during AYA well visits as intended areas of future practice change. Overall provider comfort in transitioning AYA increased significantly from matched pre-PBL to post-PBL surveys (p=0.004). Paired mean differences also showed a significant increase in provider comfort based on several identifiable skillsets in transitioning AYA. This study suggests that a formal curriculum for pediatric residents significantly increases resident comfort in transitioning AYA and encourages change in future clinical practice. Future directions include evaluating the implementation of a formal longitudinal curriculum across several PGY levels and expansion of the curriculum to include internal medicine residents. Standardized curricula on this topic may improve resident comfort on a national level.

## Introduction

Transition of care (TOC) is defined as the “purposeful, planned movement of adolescents and young adults (AYA) with chronic physical and medical conditions from child-centered to adult-oriented health care systems.” [1]. With improvements in the care of complex pediatric patients and increased life expectancy, approximately 500,000 AYA are eligible for transfer to an adult-based system every year in the USA, many of whom have special healthcare needs [2,3]. Both pediatric and internal medicine providers report little comfort in navigating barriers to the TOC process that can be identified at the provider, practice, and system level [2]. Amid a growing recognition of the importance of TOC of AYA, residency programs are increasingly tasked with educating trainees about this topic.

Although residency programs are encouraged to incorporate specific training on TOC of AYA, there is sparse research and thus limited consensus as to how this should best be accomplished [1]. Surveys of pediatric and internal medicine providers indicate that they desire a formal curriculum on transitioning to adulthood, specifically a longitudinal curriculum, due to low comfort levels [4-6]. A few residency programs have implemented formal curricula focused on transitioning AYA with neurodevelopmental disorders, disabilities, or specific chronic disease states such as cystic fibrosis, sickle cell and Type 1 diabetes [5-7]. However, in our literature search to date, we have not found a formal longitudinal transitional care curriculum with a primary care focus.

In response, we have created a longitudinal curriculum aimed at training pediatric residents about aspects of TOC of AYA in the primary care setting. This paper describes the first phase of the longitudinal curriculum which was delivered to residents in the first post-graduate year of their program (PGY1). Residents engaged in a three-pronged curriculum, with 1) a self-paced didactic on TOC, 2) clinical encounters with AYA where topics pertaining to TOC were discussed, and 3) a self-reflective exercise. The goal of this curriculum was to improve resident knowledge, familiarity, and comfort in the process of transitioning AYA.

## Materials and methods

This study was determined to be exempt from human subject research by the University of Maryland Baltimore Institutional Review Board (approval HP-00079757). Subjects included PGY1 categorical Pediatric, combined Internal Medicine and Pediatrics (Med-Peds), and combined Pediatrics and Emergency Medicine (Peds-EM) residents from the 2017-18, 2018-19, and 2019-20 academic years. Subjects participated in the AYA TOC curriculum at the University of Maryland Medical Center in Baltimore, Maryland, and completed: 1) pre-curriculum surveys, 2) a self-paced PowerPoint, 3) Got TransitionTM Readiness Assessments with one to three patients, 4) problem-based learning (PBL) exercises, and 5) post-curriculum surveys. Each subject was assigned a unique coded identifier, allowing the research team to link survey responses while maintaining anonymity. 

The pre-curriculum survey consisted of three baseline questions focused on prior training, exposure, and experience in transitioning AYA, as well as 10 questions evaluating resident comfort in several aspects of transitioning patients (Table 1). This included comfort in working with AYA ready to begin the transition process, discussing TOC with AYA and families, discussing specific differences between pediatric and adult health care models, and the ability to access resources on transitioning patients both with and without chronic conditions to provide to families, modeled after principles in the Got Transition^TM^ Initiative and joint consensus physician organization statements [8,9]. Residents were surveyed on their comfort with identifying patient factors needed for successful transition, including patient readiness, required patient skillsets, and barriers to transitioning. Comfort levels were identified using a Likert Scale from 0 to 100 (0=cannot do at all, 50=moderately certain can do, 100=certainly can do).

**Table 1:** Pre- and Post-Curriculum Survey Questions Surveys were matched at the participant level.

Have you ever had formal training on transitioning pediatric patients to the adult setting, either in medical school or residency?	Yes	No
Have you been involved in transitioning pediatric patients to the adult setting during your residency training thus far?	Yes	No
Have you been exposed to different models of transitional care?	Yes	No

Please rate your degree of confidence in performing the following activities by recording a number from 0 to 100 using the scale below	0	10	20	30	40	50	60	70	80	90	100
I am able to take care of adolescent patients who are ready to transition to an adult-based healthcare system.											
I am able to discuss the importance of transitioning to adult-based providers with my adolescent patients.											
I can identify the major barriers facing my adolescent patients as they transition to an adult-based healthcare system.											
I can discuss the major differences between the pediatric and adult-based healthcare systems.											
I can identify patient and family perceptions regarding transitioning to an adult-based system.											
I can identify the skills that my adolescent patients need to be able to transition to an adult-based healthcare system.											
I can access and use resources available to my patients regarding transitions of care.											
Specifically, I can access resources available for transitioning youth with special healthcare needs.											
I am able to assess adolescent patient’s readiness to transition.											
I am comfortable transitioning pediatric patients to the adult setting.											

Participating residents reviewed a self-paced 10-minute PowerPoint presentation (Table 2) that introduced relevant TOC topics such as an introduction on basic definitions of transition-of-care versus transfer-of-care. The PowerPoint highlighted potential barriers to TOC such as patient, family, and practitioner perceptions and differences between pediatric- and adult-centered models of care. The presentation also provided methods by which practitioners can address TOC with AYA.

**Table 2:** Curriculum: PowerPoint Presentation Text Self-paced didactic portion of curriculum. Sound bytes not included

Slide	Topic	Slide Text
1	Introduction	
2	What are Transitions of Care?	Transition is defined as the planned and purposeful movement of adolescents and young adults (AYA) from pediatric to adult health care centers by attending to their medical, psychosocial, and educational needs (1)
3	Definitions	Transition of care is different from the transfer of care. Where transition is a fluid and ongoing process, transfer is a moment in time and specifically the change in location of care (1)
4	Why do we care?	Patients, especially those with chronic conditions, tend to have worse medical and psychosocial outcomes. Transitioning patients may improve disease knowledge and self-efficacy (2), with hopes of ultimately improving outcomes as an adult.
5	The transitional period is riddled with difficulties	Pediatrics: Family centered, Informal, High level of parental involvement, High level of psychosocial support, Providers: worry that adult providers won’t care Medicine: Patient centered, Business like, sometimes less equipped to handle psychosocial support, Requires patient to function independently, More emphasis on disease, procedures, labs, Providers: believe that pediatricians are babying the patients
6	The transitional period is riddled with difficulties	Patient Perceptions: Am I ready to grow up, Why is my pediatrician abandoning me, I’m not ready to leave, Will my new doctor understand me? Parent Perceptions: Will my child get the same level of care, Will I be included, Will the medicine team understand my child the way the pediatric team does?
7	Pediatricians: How do I go about approaching transitions of care?	Introduce the topic early (as young as age 12), keeping in mind that this may need to be adjusted based on the individual patient’s maturity level. Graphic: Health Care Transition Planning Algorithm for all Youth and Young Adults Within a Medical Home (3)
8	Pediatricians: How do I approach transitions of care (3,4)	Assess for transition readiness (https://www.gottransition.org/). Plan a longitudinal process in accomplishing specific and realistic goals: Self-management skills (ex. I know how to go about filling my own prescriptions), Allocation of responsibility (ex. My parent reminds me when to take my medications), Perceived disease/regimen knowledge (ex. I can name all my medications), Demonstrated skills (ex. I can follow the instructions about a new medication/dose), Psychosocial adjustment (ex. I realize that I need my medications for the rest of my life) Implement the plan – empower and increase the responsibility of the patient over time Document the ongoing process and reassessments – it serves as a medical summary when the time to transfer approaches (you can also code for it!)
9	Pediatricians: Pre-Transfer	Make the expectations clear: when transfer will occur (“our office does not see patient’s past the age of 18”, what is expected post-transfer (“will not be seen if more than 15 mins late”), etc. For patient’s with complex chronic care, initiate discussions about guardianship and the family role in medical decision making - this is not an appropriate discussion to leave for the new provider.
10	Transfer of Care	Ideally, a “warm handoff” will occur between pediatric and adult providers – ie the providers will meet with the patient all together pre-transfer. There is some evidence that meeting the adult providers prior to transfer may help facilitate access to care (5). A written medical summary that provides the following: Baseline functional, neurological, cognitive status, Condition-specific emergency treatment plans and contacts, Patient’s health education history, current understanding of health condition, treatments, prognosis, Advanced directive (decision maker, guardianship, etc.), Patient communication preferences (interpreter, communication device, etc.).
11	Internists’ Barriers	A survey of internal medicine providers showed that often, they were uncomfortable with congenital and childhood conditions (6). Ask for help! It may be your patient now, but the pediatric provider may be able to help you troubleshoot. Don’t be afraid to ask for adult specialists’ input. Be patient – remember that this is as new to the patient as it is for you. Depending on maturity level of patient, you may be helping the patient navigate adulthood and the responsibilities that come with it. Family can be a great resource – they know a lot about the condition! Put the transfer in a positive light – “graduation”. Gently remind family that it takes time to build a relationship – and that they had to build that relationship with the pediatric provider once upon a time as well.
12	References	(1) Moreno MA. Transition of Care from Pediatric to Adult Clinics. JAMA Pediatr. 2013;167(7):684. (2) Campbell F, Biggs K et al. Transition of care for adolescents from pediatric to adult health services. Cochrane Database of Systemic Reviews 2016; (4). Doi 10.1002/14651858.CD009794.pub2. (3) Tepper, Vicki. “Transitioning from Pediatric to Adult Care”. Lecture, Pediatric Residency Program. Oct 27, 2014. Baltimore, MD. (4) American Academy of Pediatrics, American Academy of Family Physicians, American College of Physicians, Transitions Clinical Report Authoring Group. Supporting the health care transition from adolescence to adulthood in the medical home. Pediatrics. 2011;128: 182-200. (5) Fredericks EM, Dore-Stites D, Well A, et al. Assessment of transition readiness skills and adherence in pediatric liver transplant recipients. Pediatric Transplant. 2010;14(8):944–953. doi:10.1111/j.1399- 3046.2010.01349.x (6) Bloom S, et al. Health Care Transition for Youth with Special Health Care Needs. Journal of Adolescent health. 2012; 51 (3): 213-219.

**Figure 1:**
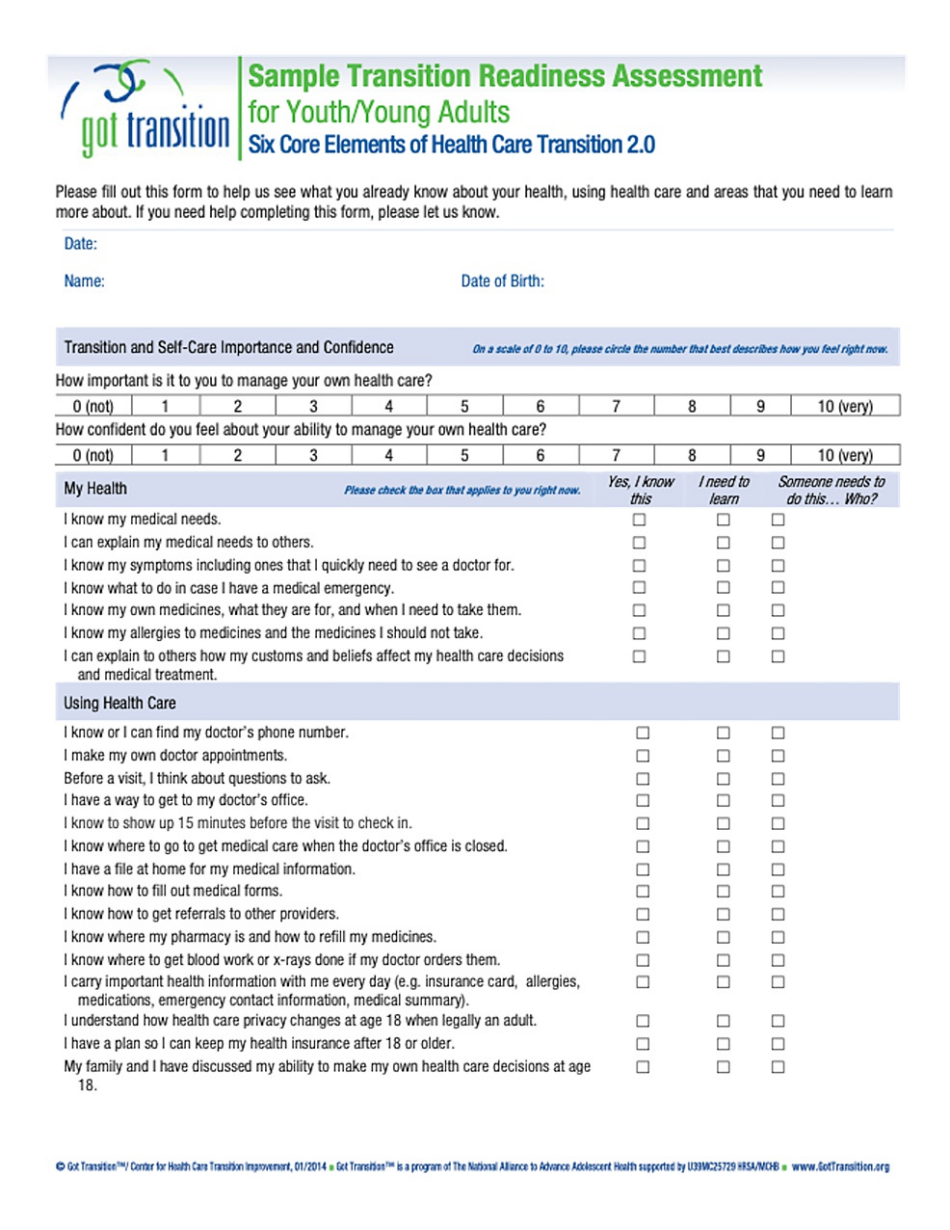
Got TransitionTM Transition Readiness Assessment White P, Schmidt A, Shorr J, Ilango S, Beck D, McManus M. Six Core Elements of Health Care Transition™ 3.0. Washington, DC: Got Transition, The National Alliance to Advance Adolescent Health, July 2020. Residents utilized the assessment during their clinical encounter with AYA patients.

Residents were asked to identify between one and three AYAs with chronic conditions during routine clinic visits at the pediatric and adolescent medicine primary care clinic, and to then incorporate a discussion on TOC to adult-based systems using the Got Transition^TM^ Readiness Assessment [8] (Figure 1). Based on the Got Transition^TM^ Readiness Assessment, AYA and resident physicians together established three goals for the AYA to work towards such as learning names and purposes of medications or calling to make one’s own appointment.

Residents then completed and submitted a PBL exercise which consisted of free text responses assessing change in perception on the importance of transitioning AYA, usefulness of the PBL, barriers to transitioning AYA, and plans to change clinical practice (Table 3).

**Table 3:** Problem-Based Learning Exercise Residents completed the problem based learning exercise (PBL) after completion of the didactic and clinical encounter portions of the curriculum.

Did this exercise change your perceptions about the importance of discussing transitional care at adolescent health supervision visits?
How did this exercise increase your awareness of barriers to transitional care?
Was the transition readiness assessment useful in identifying the specific skills that your patient needs to acquire in order to successfully transition to an adult-based system? Was your patient more or less prepared to transition than you expected?
How will this PBL exercise affect your practice? What additional training do you need in order to effectively transition your patients to an adult setting?

Residents received a post-curriculum survey identical to the pre-curriculum survey six to 12 months after introduction to the curriculum (Table 1). Participating subjects used a de-identified code to complete a pre- and post-curriculum survey and responses were matched at the participant level. Pre-curriculum responses were compared to matched post-curriculum data using the paired T-test to evaluate changes in resident self-assessed comfort after participating in the curriculum. Results were presented as mean and standard deviation (SD) and the mean (SD) difference in score was presented. Participants also completed one post-participating survey on their perception of the utility of the PBL (non-paired data) and the resulting binary and categorical data were compared using Chi Square or Fisher Exact Test as appropriate. P-values <0.05 were considered significant. Analyses were performed using SAS 9.4 (SAS Institute Inc., Cary, NC, USA).

## Results

A total of 63 PGY1 residents were eligible during the three academic years from 2017-2020 to participate in the longitudinal curriculum, of whom 22 (35%) completed both pre- and post-curriculum surveys and 35 (56%) completed the PBL exercise. Subjects who completed only one of the pre- or post-surveys were excluded. At baseline, of those who participated, two residents (9%) were exposed to formal curricula and/or various AYA TOC models prior to our intervention.

**Figure 2:**
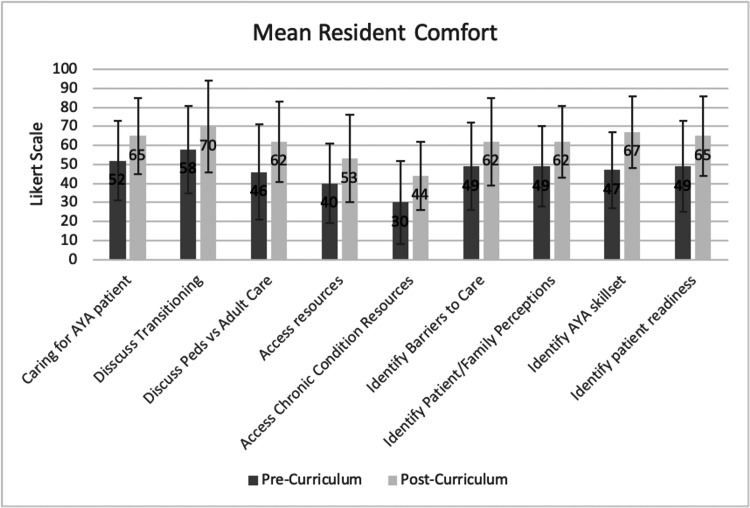
Resident Confidence in Self-Ability from Pre- to Post-Curriculum Surveys AYA: adolescents and young adults

Comfort in resident self-ability significantly improved in several measures from pre- to post-curriculum, including discussing the differences between pediatric- and adult-centered models of care (p=0.02), accessing resources pertaining to transition of AYA (p=0.006), and more specifically accessing transition resources for chronic conditions (p=0.019) (Figure 2). Residents reported significantly increased comfort in identifying patient factors necessary for successful transition of AYA patients such as barriers to care (p=0.027), patient transition readiness (p=0.017), and skills required of patients prior to transfer (p=0.0004) (Table 4). Comfort in identifying factors affecting the transition and transfer processes such as identifying family/patient perceptions on the differences between pediatric- and adult-centered care also increased significantly in the post-curriculum period (p=0.008). Overall comfort in transitioning AYA patients significantly increased from pre-curriculum to post-curriculum (p=0.004). Resident comfort in discussing the importance of transitioning with AYA did increase in our cohort, though this did not reach statistical significance (p=0.071). There was no significant difference in resident reported self-ability to care for AYA patients ready for the transitioning process.

**Table 4:** Resident Confidence in Self-Ability from Pre- to Post-Curriculum Surveys SD: standard deviation, AYA: adolescents and young adults, TOC: transition of care

Resident Comfort (N=22)	Pre-Curriculum Mean (SD)	Post-Curriculum Mean (SD)	Paired Difference (SD)	p-value
Care for transition-ready AYA patient	52 (21)	65 (20)	+7 (24)	0.1655
Discuss importance of transitioning	58 (23)	70 (24)	+ 9 (22)	0.0713
Discuss differences between pediatric and adult healthcare systems	46 (25)	62 (21)	+16 (30)	0.0200
Accessing resources for TOC	40 (21)	53 (23)	+17 (27)	0.0064
Accessing resources specifically for TOC with chronic conditions	30 (22)	44 (18)	+15 (28)	0.0186
Identify barriers to care	49 (23)	62 (23)	+14 (28)	0.0274
Identify patient and family perceptions of pediatric vs adult health care	49 (21)	62 (19)	+16 (25)	0.0078
Identify AYA patient skills necessary for transitioning	47 (20)	67 (19)	+21 (24)	0.0004
Assess patient readiness for transitioning	49 (24)	65 (21)	+15 (30)	0.0167
Overall comfort in transitioning AYA patients	44 (23)	63 (23)	+22 (32)	0.0043

**Figure 3:**
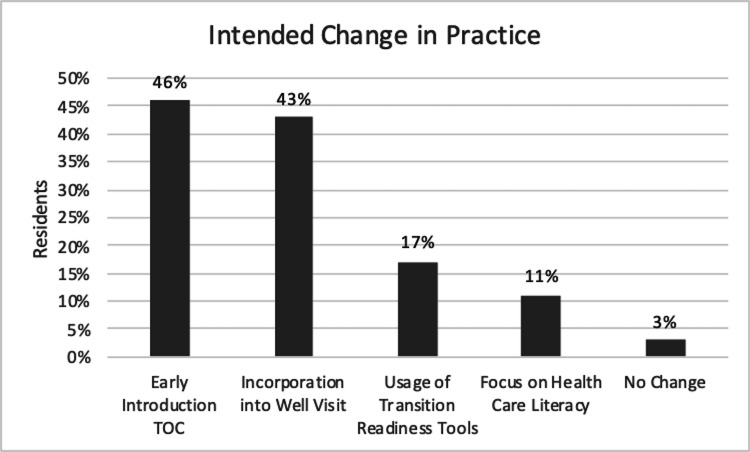
Resident reported intended change in practice on the PBL. PBL: problem-based learning, TOC: transition of care

Thirty-five residents completed the free-form short answer PBL. Eighty-six percent (n=30) identified that the PBL exercise changed their perception on the importance of introducing TOC topics during AYA clinic visits. Of the 33 residents who responded to the question on utility of the PBL, 94% (n=31) identified the PBL as being overall useful. No differences were noted between annual PGY1 cohorts (Table 5). After completion of the PBL, most residents designated more than one intended change in clinical practice. These included earlier introduction of the topic of TOC to patients (n=16, 46%), incorporation of the topic during routine visits with patients (n=15, 43%), usage of transition-readiness assessments (n=6, 17%), and focusing on health literacy (n=4, 11%) (Figure 3).

**Table 5:** Resident Perception of Problem-Based Learning (PBL) and Patient Transition Readiness by Year ^1^Survey of residents on perception of PBL after participation in the curriculum. ^2^2 of 13 residents did not respond to the question  “Was PBL useful” during 2018-2019 cohort. Denominator for 2018-2019 cohort for PBL usefulness is 11, and for total N answering question was 33.

Resident Perception^1^	2017-2018 (N=16)​ n (%)	2018-2019 (N=13)​ n (%)	2019-2020 (N=6)​ n (%)	P-Value​	Total​ (N=35) n (%)
The PBL was useful.^2^	15 (94)​	10 (91)​	6 (100)​	0.7536	31 (94)​
The PBL changed the perception on introducing transition of care to patients.	13 (81)​	11 (85)​	6 (100)​	0.4299	30 (86)​
Expected patient transition readiness:				0.6415	
More than expected	8 (50)	4 (31)	1 (17)		13 (37)
Equivalent	3 (19)	3 (23)	2 (33)		8 (23)
Less than expected	5 (31)	6 (46)	3 (50)		14 (40)

**Figure 4:**
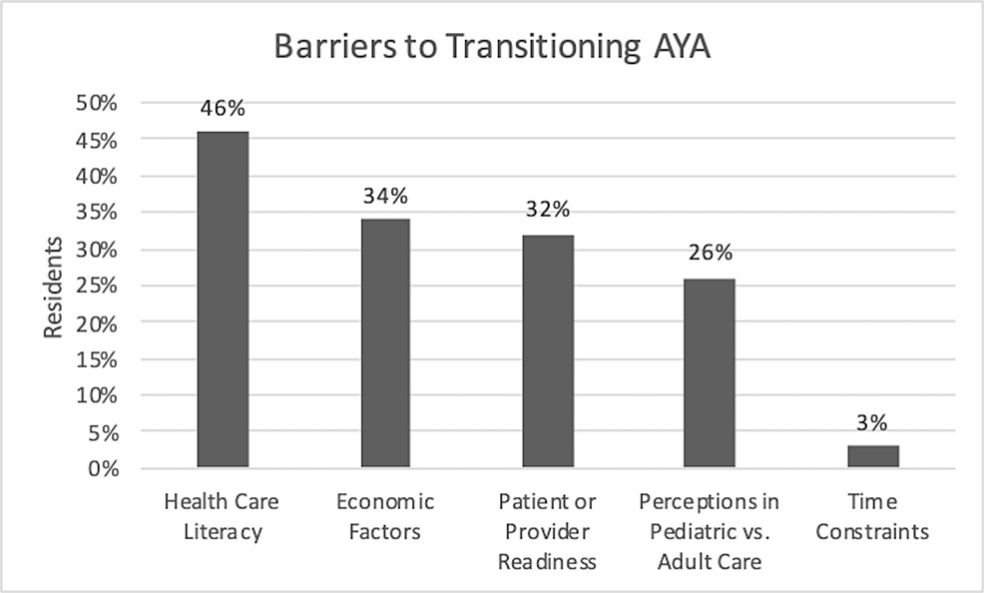
Resident reported barriers to transitioning AYA as identified on PBL AYA: adolescents and young adults, PBL: problem-based learning

Through the PBL exercise, residents identified one or more barriers in transitioning care of AYA patients, including healthcare literacy (n=16, 46%), socioeconomic factors (n=12, 34%), provider or AYA/family perception on differences in pediatric versus adult care (n=9, 26%), and time constraints (n=1, 3%) (Figure 4). Healthcare literacy barriers included knowledge of medical history and medications, access to medical records, and skills required for adequate care such as making appointments. Barriers in the form of socioeconomic factors included navigating insurance issues, access to adequate finances and transportation, and housing instability. Thirty-two percent (n=11) further identified lack of patient readiness or lack of provider readiness (discomfort with transitioning those with chronic conditions, being unaware of adult resources to provide to patients) as barriers to transitioning patients (Figure 4). Patient readiness as gauged by residents was not significantly different across cohorts (p=0.64) (Table 5).

When surveyed regarding gaps in the curriculum, 40% (n=14) of the residents requested resources for patients such as printed handouts related to TOC, lists of adult providers, and insurance information. Thirty-one percent (n=11) also requested further information for themselves such as transitioning patients with specific health conditions, how to engage in a warm handoff, and improving knowledge on insurance.

## Discussion

Though transitioning care of AYA from pediatric-centered to adult-oriented health care systems is of the utmost importance, consensus on the ideal curriculum to teach resident trainees about this topic is lacking. Prior studies have evaluated curricula focused on transitioning AYA with specific disorders or chronic disease. Our study is the first to evaluate a formal longitudinal transitional care curriculum with a primary care focus. After implementation of the first phase of our curriculum, results from our pilot study showed a statistically significant increase in resident comfort level in skills required to transition AYA such as the ability to discuss the transition process with patients and to identify barriers that AYA may face during the transition process.

Review of the literature shows few formal curricula addressing TOC of AYA among residency programs despite reported discomfort and desire to learn further about this topic among both pediatric and internal medicine providers [4-7]. Specifically, residency programs that do have formal curricula often target specific chronic conditions. Our TOC curriculum is intended as a basic introduction in transitioning AYA with chronic conditions in the primary care setting. With the growing need for residency programs to address TOC of AYA formally, our curriculum was kept as generalized as possible in order to aid in ease and feasibility of disseminating and applying the curriculum broadly across various residency institutions.

In addition to statistically significant increased individual comfort level in the process of transitioning AYA after implementation of the curriculum, residents self-identified areas of intended change in future clinical practice. Though we did not study patient outcomes, we hope that increased provider comfort and identifiable ways of improving future clinical practices may impact patient outcomes around the transition and transfer periods in favorable ways.

The research team anticipated time constraint to be a large barrier in initiating the discussion of transitioning AYA, but only one resident identified this as a barrier. These findings suggest that introducing and discussing the topic of TOC is plausible during a routinely scheduled AYA visit, even with often packed clinic schedules.

The only measure that did not significantly increase was resident comfort in ability to care for a transition-ready AYA patient, though overall comfort in transitioning patients did significantly increase. We suspect that residents were not aware of the difference in nomenclature of transitioning patients versus transferring patients prior to participating in the curriculum and that may have played a role in how residents answered pre-curriculum vs post-curriculum surveys.

Our study has several limiting factors. First, there is a small sample size due to incomplete resident participation, with only ~50% for the 2017-18 and 2018-19 cohorts completing all aspects of the curriculum. The 2019-20 cohort had even less participation due to impact of COVID-19 on clinical rotations. Our results therefore may not reflect the views of our entire resident cohort. The study was also conducted at a single urban academic center and may not be generalizable to all residency programs. Third, post-survey responses were delayed by six to 12 months from the time of introduction of the curriculum and may reflect increased comfort due to the clinical nature of residency rather than a formal curriculum. Responses from former graduates of the program who did not participate in a formal curriculum on AYA TOC will serve as a baseline to assess for difference in timing in ongoing analyses of this curriculum. Lastly, our residents evaluated the educational aspect of the curriculum rather than evaluating the process of transitioning AYA itself and therefore cannot be used to gauge if the curriculum has positive effects on patient outcomes.

We will continue to expand on our curriculum in order to address the reported discomfort in the literature among both pediatric and internal medicine providers on TOC of AYA [4-6]. The second phase of the curriculum will include following up with patients to see if they have made progress on the goals outlined during the initial visit and to gauge if the patient is progressing in the transitioning process. This will provide valuable experience for residents on how to help patients navigate the transition process. The third phase of the curriculum will simulate a warm handoff between pediatric and internal medicine residents, providing a space for both sets of residents to enhance their communication skills and practice transferring a patient’s care. Future versions of this curriculum may also include the distribution of more resources to support providers and patients navigating the difficult transitioning process, as identified by our residents, while still maintaining generalizability.

## Conclusions

We developed a longitudinal curriculum focused on the TOC of AYA for pediatric residents. The first phase of the curriculum aimed at PGY1 included learning through didactic, clinical experiences, and self-reflections. Residents subsequently demonstrated significantly increased comfort across various measures of the process of transitioning patients after implementation of the first phase of our curriculum. Significant increases were seen post-curriculum overall in self-determined resident ability to transition AYA. Though our study was performed at a single academic urban institution, we hope that our curriculum can serve as a guide for curricula at other pediatric residency programs in the future.

## References

[1] Campbell F, Biggs K, Aldiss SK (2016). Transition of care for adolescents from paediatric services to adult health services. Cochrane Database Syst Rev.

[2] Peter NG, Forke CM, Ginsburg KR, Schwarz DF (2009). Transition from pediatric to adult care: internists' perspectives. Pediatrics.

[3] Sharma N, O’Hare K, Antonelli RC, Sawicki GS (2014). Transition care: future directions in education, health policy, and outcomes research. Acad Pediatr.

[4] Mennito S (2012). Resident preferences for a curriculum in healthcare transitions for young adults. South Med J.

[5] Patel MS, O'Hare K (2010). Residency training in transition of youth with childhood-onset chronic disease. Pediatrics.

[6] Nazarian BL, Glader L, Choueiri R, Shipman DL, Sadof M (2010). Identifying what pediatric residents are taught about children and youth with special health care needs and the medical home. Pediatrics.

[7] Chung RJ, Jasien J, Maslow GR (2017). Resident dyads providing transition care to adolescents and young adults with chronic illnesses and neurodevelopmental disabilities. J Grad Med Educ.

[8] White P, Schmidt A, Shorr J, Ilango S, Beck D, McManus M (2020). Six Core Elements of Health Care Transition™ 3.0. https://www.gottransition.org/6ce/?integrating-full-package.

[9] White PH, Cooley WC (2018). Supporting the health care transition from adolescence to adulthood in the medical home. Pediatrics.

